# Prevalence and genetic characterization of *Campylobacter* from clinical poultry cases in China

**DOI:** 10.1128/spectrum.00797-23

**Published:** 2023-10-17

**Authors:** Xiaofei Li, Xiangxiang Xu, Xinyi Chen, Yunlu Li, Jiale Guo, Jie Gao, Xinan Jiao, Yuanyue Tang, Jinlin Huang

**Affiliations:** 1 Jiangsu Key Laboratory of Zoonosis, Jiangsu Co-Innovation Center for Prevention and Control of Important Animal Infectious Diseases and Zoonoses, Yangzhou University, Yangzhou, Jiangsu, China; 2 Key Laboratory of Prevention and Control of Biological Hazard Factors (Animal Origin) for Agrifood Safety and Quality, Ministry of Agriculture of China, Yangzhou, Jiangsu, China; 3 Joint International Research Laboratory of Agriculture and Agri-product Safety, Ministry of Education of China, Yangzhou, Jiangsu, China; Universidad Andres Bello, Santiago, Chile

**Keywords:** *Campylobacter *spp., prevalence, diseased poultry, China

## Abstract

*Campylobacter* is one of the leading causes of bacterial foodborne diseases globally. Poultry is considered a major reservoir for the transmission of *Campylobacter* to humans. The occurrence and epidemiology of *Campylobacter* in healthy poultry have been extensively studied, but the prevalence in diseased poultry has rarely been reported. In this study, a total of 1,563 intestinal samples, 547 gallbladder samples, and 3,729 parenchyma organ samples were collected from diseased chickens, ducks, and geese in Yangzhou Veterinary Hospital between December 2016 and September 2017. The bacterial isolates were analyzed for genotype, resistance, and virulence genes. The overall prevalence of *Campylobacter* was 17.9%, and a significantly higher prevalence was observed in the diseased chickens (21.8%) than in the diseased ducks (12.1%) and geese (11.9%). *Campylobacter* was significantly more prevalent in the intestinal tract (39.6%), followed by the gallbladder (11.7%) and parenchyma organ (9.7%). Among 84 whole-genome sequenced *Campylobacter* isolates, we identified 47 multi-locus sequence typing types, of which ST-51 and ST-825 were the most predominant sequence types (STs) for *Campylobacter jejuni* and *Campylobacter coli*, respectively. Phylogenetic analysis showed that *Campylobacter* isolates from different tissues from the same host tended to cluster together with the same STs, which indicated *Campylobacter* infection in different tissues *in vivo*. The antimicrobial resistance genes most frequently identified were *gyrA* (T86I) (97.6%) and *tet (O)* (65.5%). Our results revealed the high prevalence of *Campylobacter* in diseased poultry in China, which will help the farms take the necessary action to develop effective mitigation strategies for reducing *Campylobacter* infection in poultry.

**IMPORTANCE:**

*Campylobacter* is a major cause of campylobacteriosis worldwide, and poultry is the main reservoir for its transmission. *Campylobacter* was generally considered to be a harmless commensal organism in poultry without pathogenic properties. However, it was proposed that a *Campylobacter*-like organism may be the cause of vibrionic hepatitis, which poses a significant public health risk. The occurrence and epidemiology of *Campylobacter* in healthy poultry have been studied systematically, but little is known about the epidemiology of *Campylobacter* isolates from diseased poultry in China. Therefore, this study determined the prevalence and molecular characterization of *Campylobacter* from diseased chickens, ducks, and geese in Yangzhou Veterinary Hospital between December 2016 and September 2017, which was critical for improving the diagnosis and prevention of *Campylobacter* infections.

## INTRODUCTION

*Campylobacter* is an important zoonotic pathogen and is often considered to be one of the leading causes of bacterial foodborne illness globally (1 – 3). They are microaerophilic and grow under microaerobic conditions (10% CO_2_, 5% O_2_) at 42°C (4). *Campylobacter* can cause gastrointestinal infections with symptoms of inflammatory, bloody diarrhea or dysentery syndromes, mostly consisting of cramps, fever, and pain. Occasionally, more serious complications such as arthritis, septicemia, and Guillain-Barré syndrome can also occur with infection with *Campylobacter jejuni* or *Campylobacter coli* (5, 6).

The most common cause of *Campylobacter* infections is foodborne, which is primarily caused by poultry, including chickens, ducks, and geese (1, 5, 7). Chickens dominate the world poultry industry (8). Meanwhile, the duck and goose industries are also important sectors of the poultry industry. China is the world leader in goose production, accounting for 94.1% of global production (9, 10). *C. jejuni* and *C. coli* are often found naturally in poultry without any pathogenic features, which are primarily colonized in the ceca and colon, while the small intestine is colonized to a lesser extent (6, 7, 11). Colonization naturally occurs by horizontal transmission from the environment, and the infection rapidly spreads within the flock from one bird to another (12).

However, increasing evidence shows that *Campylobacter* can invade and destroy the intestinal mucosa and invade other organs, thereby spreading to other internal tissues (13, 14). Clinical outcomes of its infection range from asymptomatic infection to life-threatening extraintestinal infections (15). For example, *Campylobacter* infections are associated with abortion in primates and ruminants by invading the genital tract (3, 16). Meanwhile, Hasel et al. observed the heavy colonization of *Campylobacter* in human kidney abscesses (4). Other studies have also found *Campylobacter* in abscess infections of the head, brain, and chest walls (4, 17, 18).

In contrast, extraintestinal infections in poultry were rarely reported. Current studies describe extraintestinal infections in chickens but not in ducks and geese (13, 19, 20). Studies have identified *Campylobacter* in the livers of birds with no signs of disease or with vibrionic hepatitis (13). The high colonization of *Campylobacter* may contribute to the formation of white spots in the livers of commercially farmed birds (13, 20). Spotted liver disease in chickens affects mortality and production, ranging from sporadic deaths in chickens to severe decreases in egg production and an increase in mortality of more than 1% per day in egg-laying flocks (13). Infection of extraintestinal organs caused by *Campylobacter* poses a significant public health risk (20). Therefore, it is important to investigate the distribution and epidemiology of *Campylobacter* isolated from diseased poultry.

Poultry diseases are known as a critical factor limiting the development of the poultry industry (21). Numerous studies on the occurrence and epidemiology of *Campylobacter* in healthy poultry have been carried out (9, 22 – 24). However, few studies have focused on the epidemiology of *Campylobacter* in diseased poultry. This study aimed to determine the molecular epidemiology of *Campylobacter* isolates from diseased poultry at Yangzhou Veterinary Hospital, China. In addition, the molecular characteristics, genetic relationship, resistome, and virulome features of *Campylobacter* isolates from the intestinal tract, gallbladder, and parenchymatous organs of diseased poultry were investigated based on whole genome sequencing (WGS) analysis. This study provides preliminary data on *Campylobacter* from diseased poultry in China.

## MATERIALS AND METHODS

### Sample collection

The source of our samples was diseased poultry from various cities collected and diagnosed at the Yangzhou Veterinary Hospital between December 2016 and September 2017. Poultry sources collected in this study suffered from bacterial, viral, or parasitic infections with symptoms such as enteritis, hepatitis, pericarditis, or other lesions concerning organs. During isolation, one sample was collected from each lesion of a diseased poultry carcass for identification. The collection niches were the tissues where the lesion occurred; for example, it could be parenchymal organs (i.e., heart, liver, spleen, lung, and kidney), gallbladder, and intestines, so the number of samples collected from each individual source varied with the status of the lesion. Thus, the prevalence calculations pertain to samples and not individual animals. The majority of poultry sources came from different farms, with an age range of 5 days to 1 year. A total of 1,563 intestinal samples, 547 gallbladder samples, and 3,729 parenchymal organ samples (i.e., heart, liver, spleen, lung, and kidney) from diseased chickens, ducks, and geese with enteritis, hepatitis, pericarditis, or other symptoms concerning organs were collected in this study. Specifically, 946 intestinal samples, 334 gallbladder samples, and 2,264 parenchyma organ samples were derived from chickens. Then, 148 intestinal samples, 48 gallbladder samples, and 357 parenchyma organ samples were collected from ducks. Additionally, 469 intestinal samples, 155 gallbladder samples, and 1,110 parenchyma organ samples were collected from geese (Table 1). All samples were collected as described previously (20). In brief, all the samples were aseptically placed into sterile Whirl-Pak bags (Nasco, Fort Atkinson, WI, USA), labeled, stored on ice, and immediately transported to the laboratory at Yangzhou University within 24 h. The experiment was strictly conducted according to the Guide for the Care and Use of Laboratory Animals of the Ministry of Health [SYXK(Su) 2017-0045], China, with the permission of the Research Ethics Committee of Yangzhou University.

**TABLE 1:** The prevalence of *Campylobacter* spp. in this study

Host	Source		*C. jejuni* (%)	*C. coli* (%)	Total (%)
Chicken		Intestinal tract (*n* = 946)	131 (13.85)	316 (33.40)	447 (47.25)
		Gallbladder (*n* = 334)	40 (11.98)	10 (2.99)	50 (14.97)
		Parenchyma organs (*n* = 2,262)	145 (6.42)	130 (5.75)	275 (12.16)
Duck		Intestinal tract (*n* = 148)	33 (22.30)	11 (7.43)	44 (29.73)
		Gallbladder (*n* = 48)	3 (6.25)	0	3 (6.25)
		Parenchyma organs (*n* = 357)	19 (5.32)	1 (0.28)	20 (5.60)
Goose		Intestinal tract (*n* = 469)	69 (14.71)	59 (12.58)	128 (27.29)
		Gallbladder (*n* = 155)	9 (5.81)	2 (1.29)	11 (7.10)
		Parenchyma organs (*n* = 1,110)	42 (3.78)	26 (2.34)	68 (6.13)

### Isolation and identification of *Campylobacter*

The isolation and identification of *Campylobacter* were performed as previously described with some modifications (3). Briefly, all samples were homogenized in phosphate-buffered saline using homogenizers and then enriched in buffered peptone water for 24 h at 42°C in a microaerobic atmosphere of 10% CO_2_, 5% O_2_, and 85% N_2_. After enrichment, approximately 10 µL of the culture was streaked onto *Campylobacter* blood-free selective agar-containing charcoal cefoperazone deoxycholate (CCDA) (Oxoid, Basingstoke, United Kingdom) containing six antibiotics (60 µg/mL cefoperazone, 10 µg/mL rifampicin, 20 µg/mL amphotericin B, 6 µg/mL polymyxin B, 10 µg/mL trimethoprim, and 100 µg/mL cycloheximide) and incubated for 48 h at 42°C under microaerobic conditions. Suspected *Campylobacter* colonies were evaluated based on morphology (gray or brown, wet with metallic luster) and then streaked onto the CCDA plates without antibiotics, which were further incubated at 42°C under microaerobic conditions. All isolates were identified by multiplex PCR using *16S rRNA*, *mapA*, and *ceuE* primers. All primers used in this study are listed in Table S1. Three to four colonies were picked up and confirmed as *C. jejuni* or *C. coli* by multiplex PCR. One colony of *C. jejuni* or *C. coli* for each sample was stored at −80°C.

### Whole genome sequencing

For strain selection, available isolates were stratified by different hosts (chickens, ducks, and geese), and we randomly selected isolates from different niches to ensure a similar representation of different hosts and different isolation sites. A total of 84 strains were selected, including 46 *C*. *jejuni* isolates and 38 *C*. *coli* isolates (Table S2). The genomic DNA of the isolates was extracted using the TIANamp Bacteria DNA Kit (TIANGEN, China) according to the manufacturer’s instructions. WGS was carried out using the NovaSeq 6000 sequencing platform (Illumina Inc., San Diego, CA, USA) (3). Quality-controlled paired-end reads were *de novo* assembled independently using the SPAdes v3.10 assembler (3). The sequence data of all isolates have been deposited in NCBI BioProject PRJNA926478 with the BioSample accession numbers SAMN32874603 to SAMN32874686 (Table S3).

### Molecular characteristics analysis

WGS data were used for multi-locus sequence typing (MLST) typing. The sequence types (STs) and clone complexes (CCs) were obtained by submitting the whole genome sequence of isolates to the *Campylobacter* MLST database (https://pubmlst.org/organisms/campylobacter-jejunicoli). Furthermore, the WGS data were used to identify antimicrobial resistance genes and virulence genes. Antimicrobial resistance determinants were screened in each *Campylobacter* genome using ABRicate, which includes the Resfinder, CARD, ARG-ANNOT, and NCBI ARRGD databases (25). Regarding macrolides, two markers of antimicrobial resistance, including the A2075G mutation in the gene encoding 23S rRNA and the *ermB* gene, were analyzed (26). *gyrA* and 23S RNA gene point mutations were determined using Resfinder (25). The *bla*_OXA605_-positive isolates were screened for the single-nucleotide mutation (transversion G→T) in the promoter region of the target gene. NCBI blast tools were used to compare nucleotide sequences (27). Virulence genes were identified using BLAST against the VFDB database (mgc.ac.cn/VFs/) (28). Core genome single-nucleotide polymorphisms of the *C. jejuni* and *C. coli* isolates were obtained and given to FastTree2 (reference) for the reconstruction of the Maximum Likelihood tree using ParSNP (29). Furthermore, the tree data were visualized and modified using interactive Tree Of Life. The tree length is ignored for the sake of easier visualization.

### Statistical analysis

For the prevalence of *Campylobacter* spp. from different poultry or niches, the data were analyzed using the chi-square test with the SPSS statistical package (SPSS Inc., Chicago, USA). Statistical significance was set at *P* ≤ 0.05.

## RESULTS

### The prevalence of *Campylobacter* isolates from diseased poultry

The prevalence of *Campylobacter* spp. in diseased poultry carcass samples is summarized in Table 1. Out of the 5,839 samples, 1,046 (17.9%) were positive for *Campylobacter* spp. By PCR analysis, 491 (46.9%) isolates were *C. jejuni* and 555 (53.1%) *C*. *coli. Campylobacter* spp. were significantly more prevalent in diseased chickens (21.8%, 772/3,542) than in diseased ducks (12.1%, 67/553) and diseased geese (11.9%, 207/1,734) (*P*＜0.05). Among the diseased duck and goose samples, the overall prevalence of *C. jejuni* was higher than that of *C. coli*. However, there were 456 *C*. *coli* isolates from diseased chicken with a ratio of 12.9% (456/3,542), which was higher than the *C. jejuni* of 8.9% (316/3,542). The prevalence of *Campylobacter* spp. isolated from the intestinal tract (47.3%) was significantly higher than that isolated from the gallbladder (15.0%) and parenchyma organs (12.2%) in diseased chickens (*P*＜0.05). Similar trends were found in diseased duck and goose hosts (*P*＜0.05).

### Molecular typing of the *Campylobacter* isolates

To gain insight into the diversity of *C. jejuni* and *C. coli* isolates from diseased poultry, 84 isolates, including 46 *C*. *jejuni* isolates and 38 *C*. *coli* isolates in this study, were subjected to whole genome sequencing. MLST analysis grouped the 46 *C*. *jejuni* isolates into 32 STs. The most common ST types were ST-51 (8.7%, 4/46), followed by ST-305 (6.5%, 3/46) and ST-2274 (6.5%, 3/46). These 32 STs were assigned to 9 CCs, with CC574 (13.0%, 6/46), CC443 (8.7%, 4/46), and CC464 (6.5%, 3/46) being the most frequent CCs. CC574 (31.6%, 6/19), CC464 (15.8%, 3/19), and CC443 (15.8%, 3/19) were the most common CCs among the *C. jejuni* isolates from diseased chickens. Meanwhile, 38 *C. coli* isolates were divided into 15 STs. The predominant STs were ST-825 (21.1%, 8/38), followed by ST-872 (15.8%, 6/38) and ST-1145 (15.8%, 6/38). Interestingly, these 15 STs were all assigned to CC828 (94.7%, 36/38).

### Phylogenetic analysis of *Campylobacter* isolates

**FIG 1:**
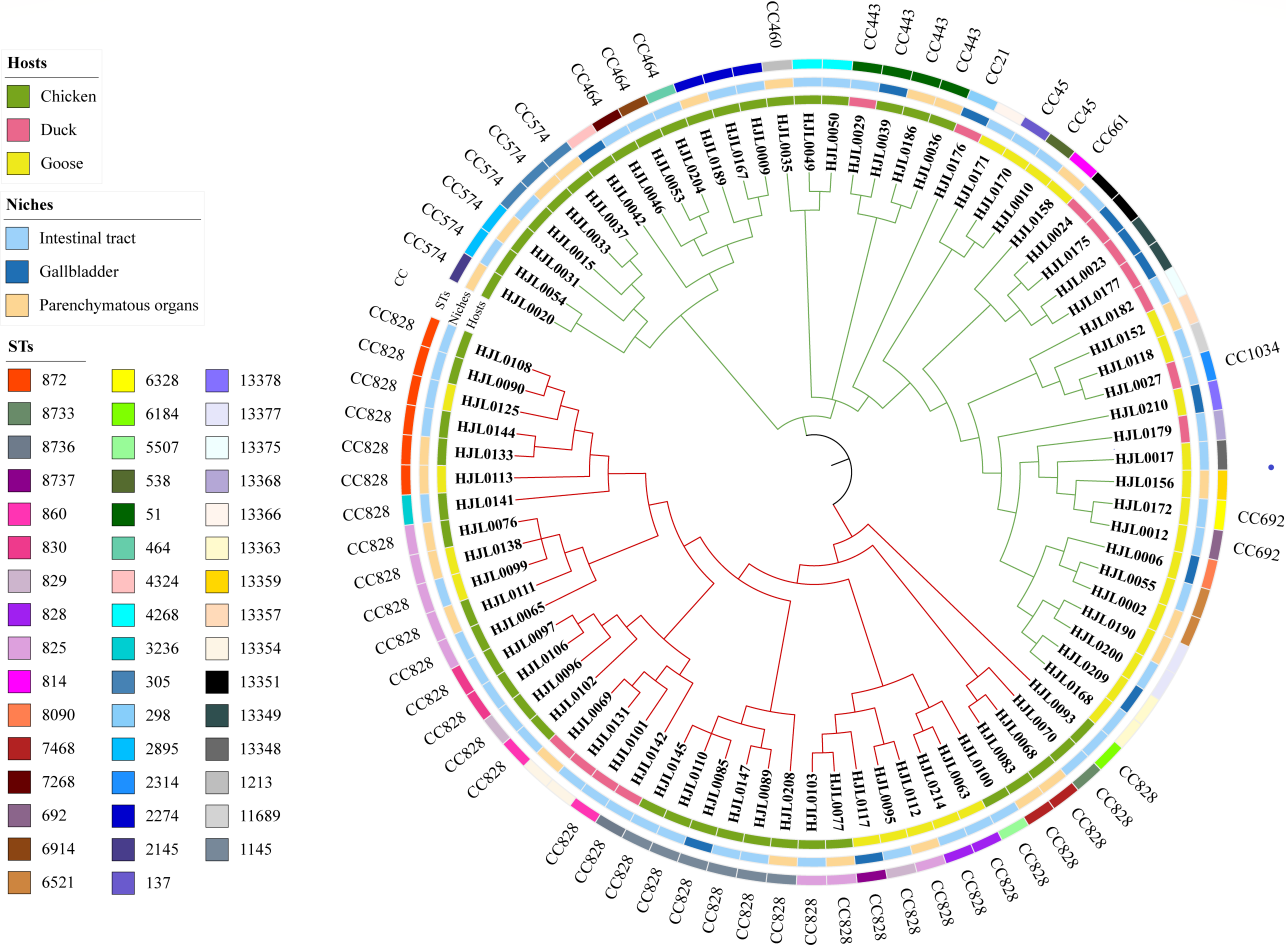
Phylogenetic tree based on the core genome and virulence genes of *Campylobacter* isolates from diseased poultry, including chickens, ducks, and geese. Eighty-four isolates were analyzed by whole genome sequencing, including 46 *C*. *jejuni* isolates and 38 *C*. *coli* isolates. *C. jejuni* isolates form the green cluster, and *C. coli* isolates form the red cluster. Visualized in the interactive Tree Of Life tool.

The phylogenetic tree was constructed using maximum likelihood estimation (Fig. 1). Phylogenetic analysis showed high genetic diversity among the *C. jejuni* and *C. coli* isolates (Fig. 1). Forty-six *C. jejuni* isolates were divided into two clades. Interestingly, isolates from diseased chickens were clustered together, indicating a close genetic relationship with the same host. Moreover, we found that the organ isolate HJL0190 and the intestinal isolate HJL0200 from the same diseased chicken individual appeared on the same branch with the same ST type (Table S2). *C. jejuni* isolates from ducks clustered together with isolates from diseased geese. Notably, 38 *C*. *coli* isolates from different hosts or tissues tended to be clustered together. *C. coli* isolates from diseased chickens were closely clustered with isolates from diseased geese. We identified three pairs of strains that were from different isolation sites of the same individual chicken, present on the same branch, with the same ST type. These strain pairs included HJL0133 and HJL0144, HJL0077 and HJL0103, and HJL0085 and HJL0110 (Table S2), which suggested a multiple organ infection *in vivo* (Fig. 1).

### Resistance gene analysis of *Campylobacter*

**FIG 2:**
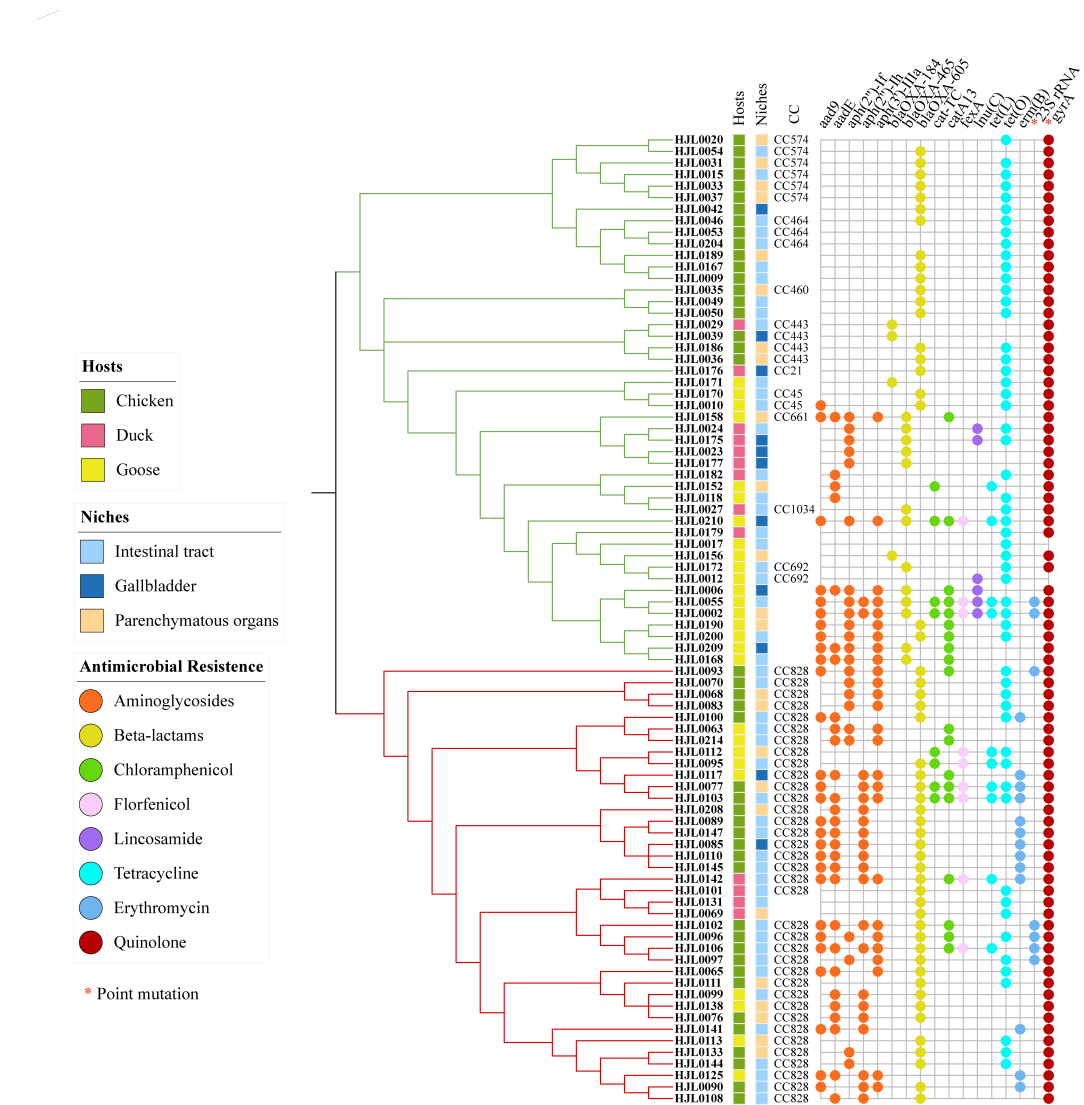
Distribution of virulence-related genes. Binary heatmaps show the presence and absence of virulence genes. *C. jejuni* isolates form the green cluster, and *C. coli* isolates form the red cluster. Colored cells represent the presence of genes.

To determine the resistome, we performed *in silico* analysis to identify genes associated with antimicrobial resistance by means of the ABRicate pipeline using the Resfinder, CARD, ARG-ANNOT, and NCBI ARRGD databases. In total, 17 antimicrobial resistance genes were detected in *Campylobacter* isolates in this study. The results are shown in Fig. 2. The point mutations in the *gyrA* gene (T86I), conferring resistance to quinolone, were the most (97.6%, 82/84) prevalent resistance gene and were detected in 44 out of 46 *C*. *jejuni* isolates and all *C. coli* isolates. The *tet(O*) gene was the second (65.5%, 55/84) prevalent resistance gene, which was detected in 36 out of 46 *C*. *jejuni* isolates and 19 out of 48 *C*. *coli* isolates. The β-lactam resistance gene *bla*_OXA-605_ was the third most (63.1%, 53/84) prevalent resistance gene and was present in 33 out of 38 *C*. *coli* isolates. The *bla*_OXA-605_ gene was more prevalent in *C. jejuni* isolates from chickens (78.9%, 15/19) than from ducks (11.1%, 1/9) and geese (22.2%, 4/18). The other two β-lactamase resistance genes, *bla*_OXA-184_ and *bla*_OXA-465_, were only present in *C. jejuni* isolates at a low frequency of 8.7% (4/46) and 28.3% (13/46), respectively. Among aminoglycoside resistance genes, *aad9*, *aadE*, *aph(2″)-lh*, and *aph(3″)-IIIa* were observed to be more prevalent in *C. coli* than in *C. jejuni*. The lincosamide resistance gene *lnu(C*) was present in 6 out of 46 *C*. *jejuni* isolates and absent in *C. coli*. Furthermore, a lower fraction of *Campylobacter* isolates carried the chloramphenicol resistance genes *cat-TC* (9.5%, 8/84) and *catA13* (11.9%, 10/84). Regarding macrolide resistance genes, the *erm(B*) gene was present in 13 out of 38 *C*. *coli* isolates and absent in *C. jejuni*. The presence of the point mutation in 23S rRNA (A2075G), responsible for macrolide resistance, was observed in 8.3% of the isolates (*n* = 84), which was detected in 2 out of 46 *C*. *jejuni* isolates and 4 out of 38 *C. coli* isolates.

### Virulence gene analysis of *Campylobacter*

**FIG 3:**
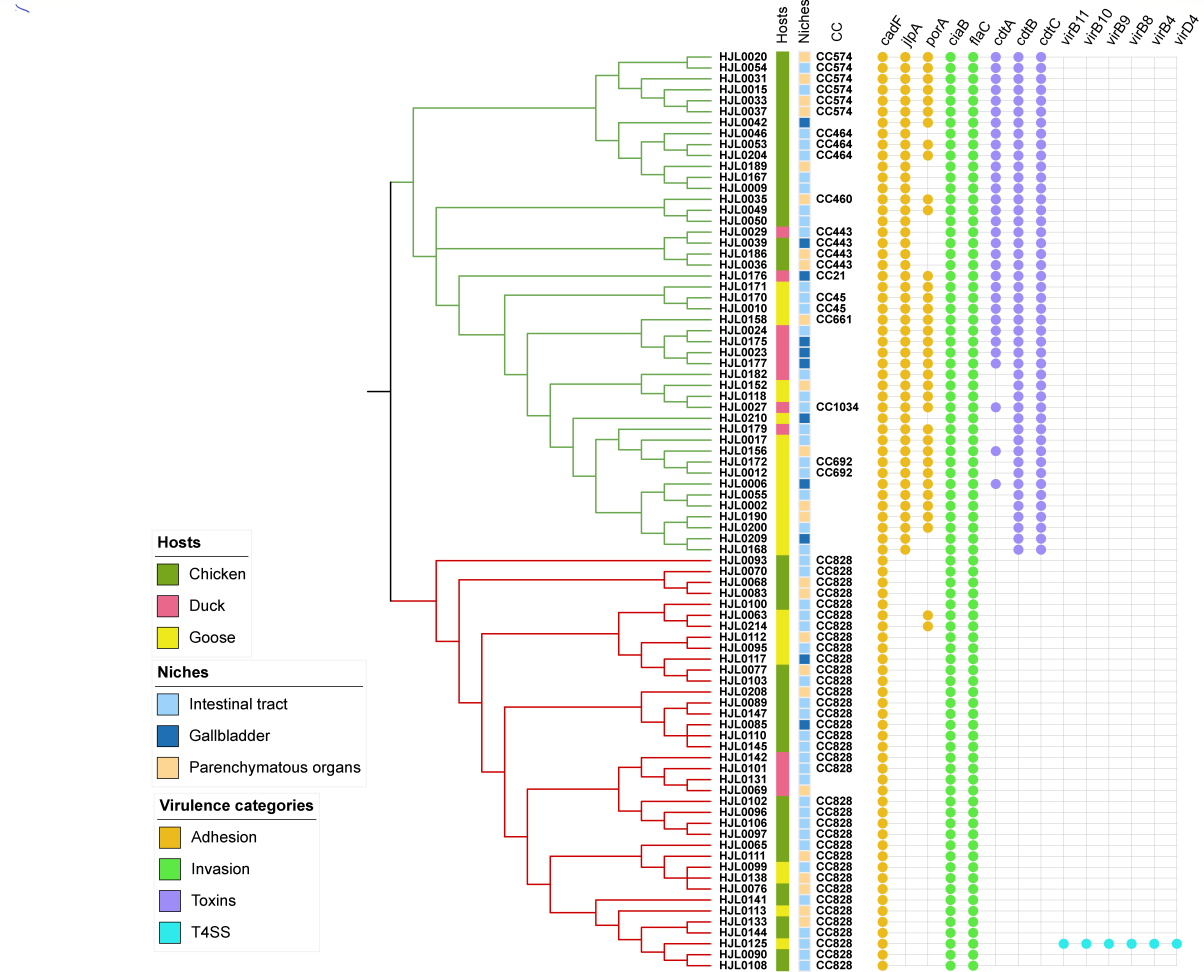
Distribution of antimicrobial resistance genes. Binary heatmaps show the presence and absence of antimicrobial resistance genes. *C. jejuni* isolates form the green cluster, and *C. coli* isolates form the red cluster. Colored cells represent the presence of genes.

The 84 whole genome-sequenced *Campylobacter* isolates in this study were tested for the presence of 14 different virulence genes, including genes relating to adhesion (*cadF*, *jlpA*, *porA*), invasion (*ciaB*, *flaC*), toxins (*cdtA*, *cdtB*, *cdtC*), and the Type IV secretion system (T4SS, *virB11*, *virB10*, *virB9*, *virB8*, *virB4*, and *virD4*). The presence of each gene in *Campylobacter* isolates is summarized in Fig. 3. *C. jejuni* isolates had more virulence genes than *C. coli* isolates, suggesting that *C. jejuni* could be more virulent than *C. coli*. For the genes associated with adhesion, the *cadF* gene was present in all *Campylobacter* isolates. Compared to *C. coli*, a higher proportion of *C. jejuni* isolates carried *jlpA* (100.0%) and *porA* (73.9%) genes for adhesion. The invasion-related genes *ciaB* and *flac* were present in all the *Campylobacter* isolates. Notably, the genes responsible for the production of the cytolethal distending toxin (CDT) (*cdtA*, *cdtB*, and *cdtC*) were found in most *C. jejuni* isolates. In contrast, they were absent in all *C. coli* isolates. Among *C. jejuni* isolated from diseased geese, 6 of 18 isolates tested positive for the *cdtA* gene. Additionally, only one *Campylobacter* isolate (HJL0125 isolate) from the intestinal tract of a goose was positive for the T4SS gene cluster.

## DISCUSSION

*Campylobacter* is an important zoonotic pathogen in humans that is prevalent in poultry. *Campylobacter* was generally considered to be a commensal organism in poultry without pathogenic properties, but little is known about the epidemiology of *Campylobacter* isolates from diseased poultry in China. Our result indicated a higher prevalence of *Campylobacter* in diseased chicken cacasses (21.8%) than in ducks (12.1%) and geese (11.9%), which was consistent with the previous observation that chickens are generally considered the most common source (30). Notably, the prevalence of *Campylobacter* in the intestinal tract of diseased chickens, ducks, and geese was higher than that in the gallbladder and parenchyma organs, suggesting that *Campylobacter* were mainly colonized in the poultry intestinal tract. Traditionally, the intestinal tract of food animals, especially poultry, is the main niche for *Campylobacter*, and the poultry intestinal tract provides a favorable environment for *Campylobacter* growth (12). However, our results showed that *Campylobacter* could also be detected in the gallbladder and parenchyma organs at a frequency ranging from 6.1% to 15.3%, implying that extraintestinal colonization by *Campylobacter* may be a reservoir for the dissemination of *Campylobacter* in poultry and humans. Laconi et al. also found *Campylobacter* in the extraintestinal tissues of chickens, including the liver and spleen (14). The consumption of chicken liver contaminated with *Campylobacter* has been associated with an increased risk of infection and has led to human campylobacteriosis (14).

In the current study, the incidence of intestinal *Campylobacter* carriage in diseased chickens was found to be 47.3%. This is higher than the prevalence ratio of 25.4% detected in the intestinal tract of healthy chickens from farms or live poultry markets in central China (31). Previous studies from both Poudel et al. and Hue et al. found that *C. jejuni* was predominant in the intestinal tract of chickens (6, 23). However, among the *Campylobacter* isolated from the intestinal tract of diseased chickens in this study, the most prevalent species was *C. coli*, accounting for 70.7% (316/447) of total intestinal *Campylobacter* isolates. This finding indicated that *C. coli* was increasingly responsible for *Campylobacter*-associated intestinal tract infections. In addition, this study shows the high prevalence of *Campylobacter* on the intestinal tract of diseased ducks (29.7%) and geese (27.3%), which agrees with the studies reported by others who found the high prevalence of *Campylobacter* spp. in ducks and geese from Iran and Poland (24, 32).

The *C. jejuni* population was highly diverse (46 isolates were assigned to 32 STs), and 50.0% of the STs were identified in isolates from chickens. This high genetic diversity observed in *C. jejuni* isolates from chickens is consistent with previous studies (33). Here, CC574 (15.2%) was the most predominant population among *C. jejuni* isolates from diseased chickens, in contrast to the result that CC21 was the most common lineage among chickens in China (34). CC443 was the second most common CC among *C. jejuni* isolates, and the third most common was CC464 (6.5%). Notably, previous studies found that isolates assigned to CC443 and CC464 were observed in both poultry and humans (7, 35 – 37), suggesting a potential bidirectional transmission of these strains between them. Our results found that 94.7% of *C. coli* isolates from diseased poultry belonged to CC828, which was reported as the predominant CC in *C. coli* from humans, poultry, and the environment (38 – 40). Interestingly, the *C. coli* isolates from chickens clustered with those from geese, and the *C. coli* isolates from different niches in the same host tended to cluster together with the same STs, suggesting potential transmissions of *C. coli* between different hosts or different niches *in vivo*.

We further found that the high percentage (97.6%) of *Campylobacter* strains with a mutation of the *gyrA* gene, which confers quinolone resistance, is consistent with previous reports indicating that *Campylobacter* exhibits a high level of resistance to fluoroquinolone (41). Regrading tetracycline resistance, 65.5% (55/84) of *Campylobacter* isolates from diseased poultry carried the *tet(O*) gene. The *tet(O*) gene has been reported to be the only tetracycline resistance determinant identified in *Campylobacter*, and it is commonly detected in all tetracycline-resistant *Campylobacter* isolates. The high prevalence of the *tet(O*) gene in our isolates suggested a potential high tetracycline resistance in *Campylobacter* isolates from poultry in China. Indeed, Han et al. confirmed that 94.6% of tetracycline-resistant *Campylobacter* isolates from broilers at slaughter in China are positive for the carriage of the *tet(O*) gene (22). The high prevalence of the *tet(O*) gene in *Campylobacter* isolates from poultry could be due to the frequent use of tetracycline during feeding.

The prevalence of the *bla*_OXA-605_ resistance gene in *C. jejuni* isolates varied among different hosts, with a higher presence in *C. jejuni* isolates from chickens (78.9%, 15/19) than from ducks (11.1%, 1/9) and geese (22.2%, 4/18). The *bla*_OXA-61_-like subfamily, which includes *bla*_OXA-605_, is reported to require promoter mutation for resistance in *Campylobacter*. The G→T transversion has been described to restore the TATA box (from GAAAAT to TAAAAT), making it fully functional, thus increasing oxacillinase production and consequently causing high-level ampicillin resistance (42, 43). In general, it was determined that 57.9% (11/19) of *C. jejuni* isolates from diseased chickens and 5.6% (1/18) of *C. jejuni* isolates from diseased geese harbored the single-nucleotide mutation. This observation prompted the formulation of a hypothesis suggesting that *C. jejuni* isolates from diseased chickens may be more resistant to β-lactams than isolates from diseased ducks and diseased geese. Mouftah et al. also found a high prevalence of the *bla*_OXA-605_ gene in *C. jejuni* from broiler carcasses (44).

The distribution of virulence genes in poultry *C. jejuni* isolates was not statistically different, suggesting that all isolates in this study had the potential to cause poultry illness. Virulence analysis showed that *C. jejuni* isolates harbored most of the known virulence factors, while *C. coli* isolates lacked most virulence genes. For example, CDT encoded by genes *cdtA*, *cdtB*, and *cdtC* plays an important role in toxin production and helps in the pathogenesis of *Campylobacter* (45). Here, we found that *cdt* gene clusters were abundant in the *C. jejuni* isolates, while they were absent in the *C. coli* isolates, indicating that *C. jejuni* is more virulent than *C. coli*.

In this study, most samples isolated from diseased poultry carcasses come from different individuals due to sampling principles, which introduces a level of individual variation that may impact the generalizability of the findings. Certainly, the fact that only 84 bacterial isolates out of a total of 1,046 isolates were randomly selected and included in the analysis presents a significant limitation to the study. This represents less than 10% of the total isolates, which could potentially impact the reliability and generalizability of the results. Further exploration is needed in the future.

### Conclusion

In conclusion, this study reveals preliminary data on *Campylobacter* from diseased poultry in China. Here, the overall prevalence of *Campylobacter* was 17.9%, and a significantly higher prevalence was observed in the diseased chickens (21.8%) than in the diseased ducks (12.1%) and geese (11.4%) (*P*＜0.05), which was consistent with the fact that chickens were the most common source. *C. coli* accounted for 53.1% of total *Campylobacter* isolates. Although *Campylobacter* was most prevalent in the intestinal tract (39.6%), it was also detected in the gallbladder and parenchyma organs of diseased poultry, with frequencies ranging from 6.1% to 15.3%, implying that extraintestinal colonization by *Campylobacter* may be a reservoir for *Campylobacter*.

Forty-seven MLST types were identified among the 84 whole genome sequences of *Campylobacter*, of which ST-51 and ST-825 were the most common STs for *C. jejuni* and *C. coli*, respectively. Phylogenetic analysis showed that *Campylobacter* isolates from different organs in the same host tended to cluster together with the same STs, suggesting a multiorgan infection of *Campylobacter in vivo*. Resistome analysis predicted widespread resistance to fluoroquinolones and tetracycline. Our results revealed the high prevalence of *Campylobacter* in diseased poultry in China, which will help to develop effective control strategies for reducing *Campylobacter* infection in poultry.

## Data Availability

The sequence data of all isolates have been deposited in NCBI BioProject PRJNA926478 with the BioSample accession numbers SAMN32874603 to SAMN32874686 (Table S3).

## SUPPLEMENTAL MATERIAL

Supplemental file 1 spectrum.00797-23-s0001.docx Tables S1 to S3.

ASM does not own the copyrights to Supplemental Material that may be linked to, or accessed through, an article. The authors have granted ASM a non-exclusive, world-wide license to publish the Supplemental Material files. Please contact the corresponding author directly for reuse.
