# Supplementary material for: Prevalence and genetic characterization of Campylobacter from clinical poultry cases in China
Source: Microbiol Spectr. 2023 Oct 17;11(6):e00797-23. doi: 10.1128/spectrum.00797-23 (PMC10714823; doi:10.1128/spectrum.00797-23)
Supplement: Supplemental file 1 — Tables S1 to S3. [file spectrum.00797-23-s0001.docx]

**Prevalence and genetic characterization of *Campylobacter* from clinical poultry cases in China**

**Table S1. Primers used in this study**

| **Genes** | **Primers** | **Sequence (5’-3’)** | **Size** | **Identification** |
| --- | --- | --- | --- | --- |
| 16S rRNA | forward | ATCTAATGGCTTAACCATTAAAC | 857 bp | *Campylobacter* genus |
|  | reverse | GGACGGTAACTAGTTTAGTATT |  |  |
| *mapA* | forward | CTATTTTATTTTTGAGTGCTTGTG | 589 bp | *Campylobacter jejuni* |
|  | reverse | GCTTTATTTGCCATTTGTTTTATTA |  |  |
| *ceuE* | forward | AATTGAAAAATTGCTCCAACTA | 462 bp | *Campylobacter* *coli* |
|  | reverse | TGATTTTATTATTTGTAGCAGCG |  |  |
|  | reverse | AACCCATCAACCTCACCCTT |  |  |

**Table S2. The information of sequencing strains**

| **Name** | **Species** | **Host** | **Host ID** | **Niche** | **Farm** | **Age** |
| --- | --- | --- | --- | --- | --- | --- |
| HJL0070 | C. coli | chicken | 20161202 C4 | Intestinal tract | Farm 26 | 30 days |
| HJL0083 | C. coli | chicken | 20161208 C14 | parenchymatous organs | Farm 37 | 2.5 years |
| HJL0111 | C. coli | chicken | 20161213 C11 | parenchymatous organs | Farm 44 | 4 months |
| HJL0100 | C. coli | chicken | 20161221 C4 | Intestinal tract | Farm 53 | 8 months |
| HJL0133 | C. coli | chicken | 20161222 C8 | parenchymatous organs | Farm 54 | 7 months |
| HJL0144 | C. coli | chicken | 20161222 C8 | Intestinal tract | Farm 54 | 7 months |
| HJL0189 | C. jejuni | chicken | 20170105 C5 | parenchymatous organs | Farm 65 | 1 years |
| HJL0077 | C. coli | chicken | 20170106 C11 | parenchymatous organs | Farm 68 | 4 months |
| HJL0103 | C. coli | chicken | 20170106 C11 | Intestinal tract | Farm 68 | 4 months |
| HJL0009 | C. jejuni | chicken | 20170228 C1 | Intestinal tract | Farm 89 | 35 days |
| HJL0204 | C. jejuni | chicken | 20170228 C2 | Intestinal tract | Farm 89 | 1 day |
| HJL0186 | C. jejuni | chicken | 20170327 C2 | parenchymatous organs | Farm 121 | 40 days |
| HJL0208 | C. coli | chicken | 20170414 C1 | parenchymatous organs | Farm 144 | 50 days |
| HJL0095 | C. coli | chicken | 20170503 C2 | Intestinal tract | Farm 171 | 36 days |
| HJL0042 | C. jejuni | chicken | 20170509 C7 | gallbladder | Farm 185 | 1 year |
| HJL0110 | C. coli | chicken | 20170523 C1 | Intestinal tract | Farm 220 | 1 year |
| HJL0085 | C. coli | chicken | 20170523 C1 | gallbladder | Farm 220 | 1 year |
| HJL0145 | C. coli | chicken | 20170523 C3 | Intestinal tract | Farm 220 | 1 year |
| HJL0167 | C. jejuni | chicken | 20170609 C6 | Intestinal tract | Farm 229 | 75 days |
| HJL0068 | C. coli | chicken | 20170611 C4 | parenchymatous organs | Farm 229 | 105 days |
| HJL0141 | C. coli | chicken | 20170612 C2 | Intestinal tract | Farm 238 | 76 days |
| HJL0097 | C. coli | chicken | 20170617 C7 | Intestinal tract | Farm 242 | 70 days |
| HJL0076 | C. coli | chicken | 20170703 C4 | parenchymatous organs | Farm 275 | 65 days |
| HJL0096 | C. coli | chicken | 20170725 C5 | Intestinal tract | Farm 292 | 5 months |
| HJL0108 | C. coli | chicken | 20170903 C1 | Intestinal tract | Farm 326 | 8 months |
| HJL0089 | C. coli | chicken | 20170903 C2 | Intestinal tract | Farm 326 | 8 months |
| HJL0102 | C. coli | chicken | 20170907 C2 | Intestinal tract | Farm 330 | 100 days |
| HJL0106 | C. coli | chicken | 20170907 C4 | Intestinal tract | Farm 331 | 100 days |
| HJL0093 | C. coli | chicken | 20170909 C1 | Intestinal tract | Farm 334 | 100 days |
| HJL0147 | C. coli | chicken | 20170912 C2 | Intestinal tract | Farm 339 | 5 months |
| HJL0090 | C. coli | chicken | 20170919 C2 | Intestinal tract | Farm 348 | 5 months |
| HJL0065 | C. coli | chicken | 20161205 C5 | Intestinal tract | Farm 29 | 30 days |
| HJL0015 | C. jejuni | chicken | 20161221 C3 | Intestinal tract | Farm 53 | 30 days |
| HJL0020 | C. jejuni | chicken | 20170413 C1 | parenchymatous organs | Farm 138 | 45 days |
| HJL0035 | C. jejuni | chicken | 20170506 C2 | parenchymatous organs | Farm 181 | 8 months |
| HJL0036 | C. jejuni | chicken | 20170509 C7 | parenchymatous organs | Farm 185 | 10 months |
| HJL0037 | C. jejuni | chicken | 20170511 C5 | parenchymatous organs | Farm 193 | 15 days |
| HJL0033 | C. jejuni | chicken | 20170703 C4 | parenchymatous organs | Farm 265 | 30 days |
| HJL0039 | C. jejuni | chicken | 20170715 C3 | gallbladder | Farm 278 | 60 days |
| HJL0050 | C. jejuni | chicken | 20170720 C1 | Intestinal tract | Farm 289 | 9 months |
| HJL0053 | C. jejuni | chicken | 20170725 C6 | Intestinal tract | Farm 291 | 5 months |
| HJL0054 | C. jejuni | chicken | 20170806 C2 | Intestinal tract | Farm 311 | 4 months |
| HJL0031 | C. jejuni | chicken | 20170907 C2 | parenchymatous organs | Farm 330 | 100 days |
| HJL0049 | C. jejuni | chicken | 20170912 C1 | Intestinal tract | Farm 339 | 5 months |
| HJL0046 | C. jejuni | chicken | 20170929 C1 | Intestinal tract | Farm 361 | 1 year |
| HJL0176 | C. jejuni | duck | 20161207 D13 | gallbladder | Farm 36 | 105 days |
| HJL0024 | C. jejuni | duck | 20170223 D3 | Intestinal tract | Farm 76 | 70 days |
| HJL0175 | C. jejuni | duck | 20170223 D3 | gallbladder | Farm 76 | 70 days |
| HJL0179 | C. jejuni | duck | 20170223 D5 | Intestinal tract | Farm 76 | 70 days |
| HJL0023 | C. jejuni | duck | 20170310 D1 | gallbladder | Farm 98 | 18 days |
| HJL0182 | C. jejuni | duck | 20170310 D3 | Intestinal tract | Farm 98 | 18 days |
| HJL0177 | C. jejuni | duck | 20170310 D4 | gallbladder | Farm 98 | 33 days |
| HJL0027 | C. jejuni | duck | 20170527 D2 | Intestinal tract | Farm 227 | 60 days |
| HJL0069 | C. coli | duck | 20170612 D1 | parenchymatous organs | Farm 240 | 30 days |
| HJL0131 | C. coli | duck | 20170612 D2 | Intestinal tract | Farm 240 | 30 days |
| HJL0101 | C. coli | duck | 20170626 D5 | Intestinal tract | Farm 262 | 170 days |
| HJL0142 | C. coli | duck | 20170626 D6 | Intestinal tract | Farm 263 | 70 days |
| HJL0029 | C. jejuni | duck | 20170629 D4 | Intestinal tract | Farm 265 | 20 days |
| HJL0006 | C. jejuni | goose | 20161230 G13 | gallbladder | Farm 62 | 14 days |
| HJL0172 | C. jejuni | goose | 20170225 G1 | Intestinal tract | Farm 83 | 220 days |
| HJL0190 | C. jejuni | goose | 20170311 G1 | parenchymatous organs | Farm 99 | 38 days |
| HJL0200 | C. jejuni | goose | 20170311 G1 | Intestinal tract | Farm 99 | 38 days |
| HJL0171 | C. jejuni | goose | 20170312 G3 | Intestinal tract | Farm 100 | 11 days |
| HJL0063 | C. coli | goose | 20170317 G2 | Intestinal tract | Farm 106 | 12 days |
| HJL0214 | C. coli | goose | 20170317 G3 | Intestinal tract | Farm 106 | 12 days |
| HJL0055 | C. jejuni | goose | 20170321 G11 | Intestinal tract | Farm 110 | 70 days |
| HJL0170 | C. jejuni | goose | 20170321 G13 | Intestinal tract | Farm 111 | 55 days |
| HJL0156 | C. jejuni | goose | 20170321 G3 | parenchymatous organs | Farm 109 | 85 days |
| HJL0099 | C. coli | goose | 20170321 G9 | Intestinal tract | Farm 110 | 90 days |
| HJL0113 | C. coli | goose | 20170325 G3 | parenchymatous organs | Farm 118 | 70 days |
| HJL0158 | C. jejuni | goose | 20170328 G4 | parenchymatous organs | Farm 123 | 33 days |
| HJL0152 | C. jejuni | goose | 20170413 G2 | parenchymatous organs | Farm 142 | 20 days |
| HJL0209 | C. jejuni | goose | 20170413 G4 | gallbladder | Farm 142 | 20 days |
| HJL0017 | C. jejuni | goose | 20170419 G2 | Intestinal tract | Farm 157 | 33 days |
| HJL0118 | C. jejuni | goose | 20170419 G3 | Intestinal tract | Farm 157 | 33 days |
| HJL0112 | C. coli | goose | 20170502 G1 | parenchymatous organs | Farm 169 | 17 days |
| HJL0010 | C. jejuni | goose | 20170510 G6 | Intestinal tract | Farm 190 | 5 days |
| HJL0138 | C. coli | goose | 20170517 G3 | parenchymatous organs | Farm 205 | 30 days |
| HJL0117 | C. coli | goose | 20170522 G1 | gallbladder | Farm 218 | 34 days |
| HJL0002 | C. jejuni | goose | 20170618 G1 | parenchymatous organs | Farm 255 | 15 days |
| HJL0210 | C. jejuni | goose | 20170625 G4 | gallbladder | Farm 255 | 23 days |
| HJL0012 | C. jejuni | goose | 20170627 G1 | Intestinal tract | Farm 264 | 4 months |
| HJL0168 | C. jejuni | goose | 20170718 G6 | Intestinal tract | Farm 286 | 20 days |
| HJL0125 | C. coli | goose | 20170718 G7 | Intestinal tract | Farm 289 | 20 days |

**Table S3.** **BioSample accession numbers of individual genome sequences**

| **Name** | **BioSample accession number** |
| --- | --- |
| HJL0009 | SAMN32874603 |
| HJL0042 | SAMN32874604 |
| HJL0068 | SAMN32874605 |
| HJL0070 | SAMN32874606 |
| HJL0076 | SAMN32874607 |
| HJL0077 | SAMN32874608 |
| HJL0083 | SAMN32874609 |
| HJL0085 | SAMN32874610 |
| HJL0089 | SAMN32874611 |
| HJL0090 | SAMN32874612 |
| HJL0093 | SAMN32874613 |
| HJL0095 | SAMN32874614 |
| HJL0096 | SAMN32874615 |
| HJL0097 | SAMN32874616 |
| HJL0100 | SAMN32874617 |
| HJL0102 | SAMN32874618 |
| HJL0103 | SAMN32874619 |
| HJL0106 | SAMN32874620 |
| HJL0108 | SAMN32874621 |
| HJL0110 | SAMN32874622 |
| HJL0111 | SAMN32874623 |
| HJL0133 | SAMN32874624 |
| HJL0141 | SAMN32874625 |
| HJL0144 | SAMN32874626 |
| HJL0145 | SAMN32874627 |
| HJL0147 | SAMN32874628 |
| HJL0167 | SAMN32874629 |
| HJL0186 | SAMN32874630 |
| HJL0189 | SAMN32874631 |
| HJL0204 | SAMN32874632 |
| HJL0208 | SAMN32874633 |
| HJL0015 | SAMN32874634 |
| HJL0020 | SAMN32874635 |
| HJL0031 | SAMN32874636 |
| HJL0033 | SAMN32874637 |
| HJL0035 | SAMN32874638 |
| HJL0036 | SAMN32874639 |
| HJL0037 | SAMN32874640 |
| HJL0039 | SAMN32874641 |
| HJL0046 | SAMN32874642 |
| HJL0049 | SAMN32874643 |
| HJL0050 | SAMN32874644 |
| HJL0053 | SAMN32874645 |
| HJL0054 | SAMN32874646 |
| HJL0065 | SAMN32874647 |
| HJL0023 | SAMN32874648 |
| HJL0024 | SAMN32874649 |
| HJL0027 | SAMN32874650 |
| HJL0029 | SAMN32874651 |
| HJL0069 | SAMN32874652 |
| HJL0101 | SAMN32874653 |
| HJL0131 | SAMN32874654 |
| HJL0142 | SAMN32874655 |
| HJL0175 | SAMN32874656 |
| HJL0176 | SAMN32874657 |
| HJL0177 | SAMN32874658 |
| HJL0179 | SAMN32874659 |
| HJL0182 | SAMN32874660 |
| HJL0002 | SAMN32874661 |
| HJL0006 | SAMN32874662 |
| HJL0010 | SAMN32874663 |
| HJL0012 | SAMN32874664 |
| HJL0017 | SAMN32874665 |
| HJL0055 | SAMN32874666 |
| HJL0063 | SAMN32874667 |
| HJL0099 | SAMN32874668 |
| HJL0112 | SAMN32874669 |
| HJL0113 | SAMN32874670 |
| HJL0117 | SAMN32874671 |
| HJL0118 | SAMN32874672 |
| HJL0125 | SAMN32874673 |
| HJL0138 | SAMN32874674 |
| HJL0152 | SAMN32874675 |
| HJL0156 | SAMN32874676 |
| HJL0158 | SAMN32874677 |
| HJL0168 | SAMN32874678 |
| HJL0170 | SAMN32874679 |
| HJL0171 | SAMN32874680 |
| HJL0172 | SAMN32874681 |
| HJL0190 | SAMN32874682 |
| HJL0200 | SAMN32874683 |
| HJL0209 | SAMN32874684 |
| HJL0210 | SAMN32874685 |
| HJL0214 | SAMN32874686 |
